# MMP Mediated Degradation of Type VI Collagen Is Highly Associated with Liver Fibrosis – Identification and Validation of a Novel Biochemical Marker Assay

**DOI:** 10.1371/journal.pone.0024753

**Published:** 2011-09-14

**Authors:** Sanne Skovgård Veidal, Morten Asser Karsdal, Efstathios Vassiliadis, Arkadiusz Nawrocki, Martin Røssel Larsen, Quoc Hai Trieu Nguyen, Per Hägglund, Yunyun Luo, Qinlong Zheng, Ben Vainer, Diana Julie Leeming

**Affiliations:** 1 Nordic Bioscience A/S, Herlev, Denmark; 2 Faculty of Health Science, University of Southern Denmark, Odense, Denmark; 3 Nordic Bioscience Beijing, Beijing, China; 4 Department of Systems Biology, Technical University of Denmark, Kgs. Lyngby, Denmark; 5 Department of Pathology, Rigshospitalet, Copenhagen University Hospital, Copenhagen, Denmark; University of Bergen, Norway

## Abstract

**Background and Aims:**

During fibrogenesis, in which excessive remodeling of the extracellular matrix occurs, both the quantity of type VI collagen and levels of matrix metalloproteinases, including MMP-2 and MMP-9, increase significantly. Proteolytic degradation of type VI collagen into small fragments, so-called neo-epitopes, may be specific biochemical marker of liver fibrosis. The aim of this study was to develop an ELISA detecting a fragment of type VI collagen generated by MMP-2 and MMP-9, and evaluate this assay in two preclinical models of liver fibrosis.

**Methods:**

Mass spectrometric analysis of cleaved type VI collagen revealed a large number of protease-generated neo-epitopes. A fragment unique to type VI collagen generated by MMP-2 and MMP-9 was selected for ELISA development. The CO6-MMP assay was evaluated in two rat models of liver fibrosis: bile duct ligation (BDL) and carbon tetrachloride (CCl4)-treated rats.

**Results:**

Intra- and inter-assay variation was 4.1% and 10.1% respectively. CO6-MMP levels were significantly elevated in CCl_4_-treated rats compared to vehicle-treated rats at weeks 12 (mean 30.9 ng/mL vs. 12.8 ng/mL, p = 0.002); week 16 (mean 34.0 ng/mL vs. 13.7 ng/mL, p = 0.0018); and week 20 (mean 35.3 ng/mL vs. 13.3 ng/mL, p = 0.0033) with a tight correlation between hepatic collagen content and serum levels of CO6-MMP (R^2^ = 0.58, p<0.0001) in CCl_4_- treated rats. In BDL rats, serum levels of CO6-MMP were significantly elevated compared to the levels in sham-operated animals both at 2 weeks (mean 29.5 ng/mL vs. 14.2 ng/mL, p = 0.0001) and 4 weeks (mean 33.0 ng/mLvs. 11.8 ng/mL, p = 0.0003).

**Conclusions:**

This novel ELISA is the first assay enabling assessment of MMP degraded type VI collagen, allowing quantification of type VI collagen degradation, which would be relevant for different pathologies. The marker was highly associated with liver fibrosis in two liver fibrosis animal models, suggesting type VI turnover to be a central player in fibrogenesis.

## Introduction

Liver fibrosis due to viral or alcohol-induced injury is one of the leading causes of death worldwide [1]. To date no curative treatment for liver fibrosis is available and patients are dependent on the success of inactivation or removal of the injurious agent or in the case of end-stage cirrhosis, on liver transplantation. Assessment of liver fibrosis is important to estimate the prognosis for the progression to liver cirrhosis and to determine surveillance strategies. At present, liver biopsy is the most commonly used method for fibrosis assessment, but it is invasive, associated with patient discomfort and, in rare cases, with serious complications [2]. In addition, the accuracy of liver biopsy is limited due to sampling error and significant intra- and inter-observer variability in histological staging [3], [4]. Therefore, research has focused on the evaluation of non-invasive methods for the assessment of liver fibrosis [5].

Fibrosis may be described as extensive scar formation, observed as increased deposition and abnormal distribution of extracellular matrix (ECM) components such as collagens and proteoglycans. ECM remodeling is a key process of tissue homoeostasis [6]–[8], and specific proteolytic activities are a prerequisite for a range of cellular functions and interactions during the process [9]. The specific proteolytic activities are precisely coordinated under physiological situations, with a specified sequence of events resulting in controlled tissue turnover. In pathological situations, including inflammations, fibrosis and cancer, the normal damage/repair balance is displaced [10], leading to excessive remodeling. As a consequence of this tissue turnover, there is a release of several protein degradation fragments specific for the combination of the involved proteases, the affected organ and the disease. The fragmentation results in exposure of new peptide ends (so-called neo-epitopes) to which specific antibodies can be developed. These neo-epitopes may be used for the design of molecular biochemical markers [11].

Endopeptidases such as metalloproteinases (MMPs) play a major part in the degradation of extracellular macromolecules such as collagens and during fibrogenesis the levels of MMPs increase [12], [13]. With respect to excessive proteolytic activity in the fibrous tissue, the gelatinases MMP-2 and MMP-9 have been investigated and documented to be highly regulated [12], [13]. Type VI collagen degradation yields several unique fragments, including the CO6-MMP fragment (YRGPEGPQGP). The combination of active and over-expressed MMPs and increased levels of type VI collagen poses the interesting hypothesis that a MMP-generated fragment of type VI collagen could be used as a biomarker of liver fibrosis.

Type VI collagen is a ubiquitous ECM protein. It is a heterotrimer composed of three different α-chains with short triple helical domains [14]. Following secretion into the ECM, type VI collagen tetramers aggregate into filaments and form an independent microfibrillar network in virtually all connective tissues, except for bone [15]. Type VI collagen has been identified within most tissues and it is generally accepted that it plays a role in the maintenance of tissue integrity since it participates in both cell-matrix and matrix-matrix interactions [16]. Type VI collagen interacta with many other ECM proteins, including fibronectin [17], type IV collagen [18], decorin, biglycan and type II collagen [19]. In close association with other collagen types, type VI collagen forms filaments and has therefore been described as a connecting protein [14], [20]. It is mainly found in a pericellular localization around a number of cell types – in the liver, mainly around hepatic stellate cells and around hepatocytes in fetal liver [14], [16]. In previous studies, total collagen type VI, a putative marker of mesenchymal activation, has been suggested to be an indicator of early architectural remodeling in liver fibrosis [21]–[24]. Serum levels of type VI collagen has been found to be elevated in patients with alcoholic cirrhosis [23], [24]. During fibrogenesis, the total amount of type VI collagen is increased [14], [24]–[26], and even though it represents only a minor fraction of the ECM collagens, the release to measurable products into the serum is significant, even in healthy individuals, indicating a considerable turnover [24],[27].

Several animal models for liver fibrosis have been developed, most of them in small rodents [28], each with individual strengths and weaknesses. These different rodent models are complementary as they represent different pathways to fibrosis, as also seen in human disease. Bile duct ligation (BDL) in rats has been used as a model of chronic liver injury due to its resemblance to hepatocyte damage, hepatic stellate cell activation, and liver fibrosis observed in human cholestatic liver diseases [1], [28]. Carbon tetrachloride (CCl4) is a hepatotoxin that causes acute liver injury and, when given repetitively at a low dose, induces liver fibrosis. It is a highly reproducible and robust model which is used to resemble alcoholic and non-alcoholic steatohepatitis with the consequent fibrosis and cirrhosis in humans [1], [28].

We hypothesized that specific MMP-2 and MMP-9 mediated degradation of type VI collagen was measurable in serum during liver fibrogenesis. The aim of this work was to develop a novel competitive enzyme-linked immunosorbent assay (ELISA) for measuring MMP-2 and MMP-9 mediated turnover of type VI collagen and to measure the neo-epitopes in two complementary experimental models of liver fibrosis induced by BDL or CCl4.

## Materials and Methods

### Ethics Statement

The BDL experiments were approved by the Experimental Animal Committee of the Danish Ministry of Justice and were performed according to the European Standard for Good Clinical Practice (2008/561-1450).

The CCl_4_ study was approved by the Ethical Committee of Animal Experimentation of the University of Barcelona (B-NNP-233/09) and was performed according to the criteria of the Investigation and Ethics Committee of the Hospital Clinic Universitari (Barcelona, Spain).

### Reagents

All reagents used for the experiments were standard high-quality chemicals from companies such as Merck (Whitehouse Station, NJ, USA) and Sigma Aldrich (St.Louis, MO, USA). The synthetic peptides used for monoclonal antibody production were purchased from the Chinese Peptide Company, Beijing, China.

### *In vitro* cleavage

Purified type VI collagen from human placenta (cat. no. ab7538, Abcam, Cambridge, UK) was cleaved with pro-MMP-2 or pro-MMP-9 (cat. no. 444213; 444231; Calbiochem, Merck, Whitehouse Station, NJ, USA). Fifty µg MMP-2 or MMP-9 was activated with 20 µl 1 mM 4-aminophenylmercuric acetate (AMPA) in dimethyl sulfoxide and incubated at 37°C for 3 hours. Type VI collagen was delivered dissolved in 0.5 M acetic acid. To facilitate MMP cleavage, the protein was dialyzed for two days to remove the acetic acid. The liquid was filtered to remove proteins below 10 kDa (Microcon Ultracel YM-10, cat. no. 42407, Millipore, Billerica, MA, USA). Each MMP cleavage was performed separately by mixing 100 µg type VI collagen and 1 µg of either MMP-2 or MMP-9 in MMP buffer (100 mM Tris-HCl, 100 mM NaCl, 10 mM CaCl_2_, 2 mM Zn acetate, pH 8.0). As control, 100 µg of collagen was mixed with MMP buffer alone. The solutions were incubated for 24 hours at 37°C. The cleavage reaction was stopped using 50 µM ethylenediaminetetraacetic acid (EDTA) to a final concentration of 1 µM. Cleavage was verified by visualization using the SilverXpress® Silver Staining Kit (cat. no. LC6100, Invitrogen, Carlsbad, Ca, USA) according to the manufacturer's instructions.

Pepsin cleavage was performed by mixing 100 µg type IV collagen and 1 µg of pepsin in pepsin buffer (0.2 M sodium acetate buffer, pH 4.0). The resultant enzyme/protein mixture was incubated at 37°C for 2 hours. At the designated time, 2 M Trizma Base was added to adjust pH to neutral to stop the reactions.

### Peptide identification

Peptide fragments in the *in vitro* cleaved samples were identified using liquid chromatography (LC) coupled to electrospray ionization (ESI) tandem mass spectrometry (LC-MS/MS). LC-MS samples were ultra-filtrated to remove proteins above 10 kDa, the pH was adjusted to 2.0 using formic acid, and a 4 µl sample was analyzed by LC-MS/MS. LC was performed on a nanoACQUITY UPLC BEH C_18_ column (Waters, Milford, MA, USA) using a formic acid/acetonitril gradient. MS and MS/MS were performed on a Synapt High Definition Mass Spectrometry quadruple time of flight MS (QUAD-TOF; Waters, Milford, MA, USA), with an acquisition range of 350–1600 m/z in MS and 50–2000 m/z, in MS/MS. The software “ProteinLynx Global SERVER (PLGS)” (Waters, Milford, MA, USA) was used to analyze spectra and generate peak lists. To identify peptides, MS and MS/MS data was searched against a type VI collagen (FASTA) protein database using the Mascot 2.2 (Matrix Science, Boston, MA, USA) software with the ESI-QUAD-TOF settings and carbamidomethyl (C), oxidation of methionine (M), oxidation of lysine (K) and oxidation of proline (P) as variable modifications.

The six amino acids in the N- or C-terminal of the peptides identified by MS were regarded as a neo-epitope generated by the protease in question. All protease-generated sequences were analyzed for homology and distance to other cleavage sites and tested for homology using NPS@: network protein sequence analysis (Combet C, Blanchet C, Geourjon C, Deleage G. NPS@:network protein sequence analysis. Trends Biochem Sci 2000; 25: 147–50).

### Peptide conjugation

The peptide conjugation was performed using the Maleidide Activated Immunogen Conjugation Kit (Sigma-Aldrich, MO, USA). Briefly, the cysteine-containing immunogenic neo-epitope (YRGPEGPQGP-GGC, 400 µl peptide at 5 mg/ml) with one free sulfhydryl (-SH) group was mixed in conjugation buffer with the maleimide-activated ovalbumin (OVA) (180 µl OVA at 10 mg/ml) as a carrier protein with an available maleimide group that could react with sulfhydryl-containing peptides and incubated for 2 hours at room temperature. Conjugated products were cleared of EDTA and sodium azide by desalting or dialysis for two days. For the biotin-conjugated peptides, the biotin-conjugated lysine was added in the solid-phase peptide synthesis procedure.

### Monoclonal antibody development

4–6 weeks-old Balb/C mice were immunized subcutaneously with about 200 µl emulsified antigen and 50 µg of the neo-epitope CO6-MMP (YRGPEGPQGP-GGC-OVA). Consecutive immunizations were performed at 2-week intervals until stable sera titer levels were reached in Freund's incomplete adjuvant. Blood samples were collected from the 2^nd^ immunization. At each blood sampling, the serum titer was determined and the mouse with highest anti-serum titer was selected for fusion. After the 4^th^ immunization, this mouse was rested for 1 month and then boosted intravenously with 50 µg CO6-MMP in 100 µl 0.9% sodium chloride solution three days before isolation of the spleen for cell fusion.

### Fusion and antibody screening

The fusion procedure performed as described by Gefter et al [29]. Briefly, mouse spleen cells were fused with SP2/0 myeloma fusion partner cells. The hybridoma cells were cloned using a semi-solid medium method and transferred into 96-well microtiter plates for further growth and incubated in a CO_2_-incubater. Standard limited dilution was used to promote monoclonal growth. Supernatants were screened using an indirect ELISA with streptavidin-coated microtitre plates and YRGPEGPQGP-K-Biotin as a capture peptide.

### Characterization of clones

Native reactivity and peptide binding of the monoclonal antibodies was evaluated by displacement of native samples (human/rat/mouse serum, plasma and urine) in a preliminary ELISA using 10 ng/mL biotinylated peptide coater on a streptavidin-coated microtitre plate and the supernatant from the growing monoclonal hybridoma. Specificities of the clones to a free peptide (YRGPEGPQGP), a non-sense peptide, and an elongated peptide (GYRGPEGPQG) were tested. Isotyping of the monoclonal antibodies was performed using the Clonotyping System-HRP kit, cat. no. 5300-05 (Southern Biotech, Birmingham, AL, USA). The selected clones were purified using protein G columns according to manufacturer's instructions (GE Healthcare Life Science, Little Chalfont, Buckinghamshire, UK). Selected monoclonal antibodies were labeled with horseradish peroxidase (HRP) using the Lightning link HRP labeling kit according to the instructions of the manufacturer (Innovabioscience, Babraham, Cambridge, UK).

### CO6-MMP ELISA methodology

In preliminary experiments, we optimized the reagents, their concentrations and the incubation periods by performing several checkerboard analyses. The CO6-MMP ELISA was developed as follows: A 96-well streptavidin plate was coated with biotinylated synthetic peptide YRGPEGPQGP-K-Biotin dissolved in assay buffer (25 mM Tris, 1% BSA, 0.1% Tween-20, pH 7.4) and incubated 30 minutes at 20°C. Twenty µl of peptide calibrator or sample were added to appropriate wells, followed by 100 µL of conjugated monoclonal antibody and incubated 1 hour at 20°C. Finally, 100 µL tetramethylbenzinidine (TMB) (Kem-En-Tec cat. no. 438OH) was added and the plate was incubated 15 minutes at 20°C in the dark. All the above incubation steps included shaking at 300 rpm. After each incubation step the plate was washed five times in washing buffer (20 mM Tris, 50 mM NaCl, pH 7.2). The TMB reaction was stopped by adding 100 µl stopping solution (1% HCL) and measured spectrophotometrically at 450 nm with 650 nm as the reference. A standard curve was performed by serial dilution of the CO6-MMP peptide and plotted using a 4-parametric mathematical fit model. Standard concentrations were 0, 0.39, 7.8, 15.6, 31.3, 62.5, 125 250 ng/mL.

### Technical evaluation

From 2-fold dilutions of pooled serum and plasma samples, linearity was calculated as a percentage of recovery of the 100% sample. The lower detection limit (LDL) was calculated from 21 determinations of the lowest standard (the zero standard) and calculated as the mean +3x standard deviation. The inter- and intra-assay variation was determined by 10 independent runs of 5 QC samples, with each run consisting of two replicas of double determinations of the samples. Finally, for each assay, a master calibrator prepared from synthetic peptides accurately quantified by amino acid analysis was used for calibration purposes.

The analyte stability was determined for six serum samples (three rat and three human) for 10 freeze and thaw cycles.

### ELISA characterization

The developed CO6-MMP ELISA was evaluated using 20 µl of the cleavage-samples: type VI collagen, type VI collagen cleaved with MMP-2, type VI collagen cleaved with MMP-9 described under “*In vitro* cleavage”. The negative control was *in vitro* cleaved type VI collagen with fibroblast activation protein (FAP). Cross-reactivity was tested using intact or *in vitro* cleaved type I or IV collagen using 20 µL peptide solution of 1000 ng/mL for each test in the assay. Neo-epitope specificity was tested using cleaved (by either MMP-2 or MMP-9) and non-cleaved type VI collagen and by an elongated CO6-MMP amino acid sequence (GYRGPEGPQG).

### Bile duct ligation

A total of 40 female Sprague-Dawley rats aged 6 months were housed at the animal research facilities at Nordic Bioscience, Denmark. The rats were kept in standard type III-H cages at 18–22°C with bedding and nest material (Altromin 1324; Altromin, Lage, Germany) and water *ad libitum*. Rats were kept under conditions of a 12-hour light: dark cycle. Experiments began after 1 week of acclimatization. Bile duct ligation (BDL) was performed in anaesthetized rats by ligation of the bile duct in two places and dissection between the ligations in an open-surgery procedure. In sham-operated rats, the abdomen was closed without BDL. BDL- or sham-operated rats were sacrificed after 2 or 4 weeks.

### CCl_4_ inhalation

The study included 52 3-months old male Wistar rats treated with CCl_4_ and 28 Wistar control rats (Charles-River, Saint Aubin les Elseuf, France). Complete details of the study are described elsewhere (Segovia-Silvestre T et al.). Liver damage was induced as previously described [30], and in short included administration by inhalation of CCl_4_ twice weekly. Phenobarbital (0.3 g/l) was added to the drinking water. Animals were stratified into groups receiving 8, 12, 16 or 20 weeks of CCl_4_ (n = 13 for CCl_4_; n = 7 control for each group). Control rats received Phenobarbital only. Four animals from the CCl_4_ groups died during the study. After the stated weeks of CCl4 administration the rats were weighed, anesthetized with pentobarbital (50 mg/kg) and terminated by decapitation.

### Blood and tissue sampling

Blood samples were taken under light CO_2_/O_2_ anesthesia at baseline and at termination from the retro-orbital sinus of rats which had fasted for at least 14 hours. The collected blood was left for 30 min at room temperature to clot, followed by centrifugation at 3000 *g* for 10 min. All clot-free liquid was transferred to new tubes and centrifuged again at 3000 *g* for 10 min. The serum was then transferred to clean tubes and stored at −80°C.

Livers were carefully dissected, weighed, fixed in 4% formaldehyde for a minimum of 24 hours, cut into appropriate slices and embedded in paraffin. Liver sections (4–5 µm thick) were stained with 0.1% Sirius Red F3B (Sigma-Aldrich, St. Louis, MO) in saturated picric acid (Sigma-Aldrich).

### Histology image analysis

Relative fibrosis area (expressed as a percentage of total liver area) was assessed by analyzing 36 fields of Sirius Red-stained liver sections per animal. Each field was acquired at 10× magnification [E600 microscope (Nikon) and RT-Slider SPOT digital camera (Diagnostic Instruments, Inc., Sterling Heights, Michigan, US)]. Results were analyzed using a computerized Bioquant Life Science morphometry system. To evaluate the relative fibrosis area, the measured collagen area was divided by the net field area and then multiplied by 100. Subtraction of vascular luminal area from the total field area yielded the final calculation of the net fibrosis area. From each animal analyzed, the amount of fibrosis as a percentage was measured and the average value presented [31].

### Immunohistochemistry

Liver sections (4 µm) were de-paraffinised, hydrated and further peroxidase activity was blocked with the addition of 0.4% hydrogen peroxide. Sections were then incubated with a polyclonal antibody against type VI collagen (1∶100; Abcam, Cambridge, UK). Sections were then rinsed and the antibody binding was depicted using the Super Sensitive Polymer-HRP IHC Detection System combined with AEC substrate, according to the supplier's instructions (Biogenex, Taby, Sweden). Sections were counterstained with Mayer's haematoxylin. Digital photographs were taken using an Olympus B×60 microscope with ×40 magnification and an Olympus 5050-zoom digital camera (Olympus, Tokyo, Japan).

## Results

### *In vitro* cleavage and selection of peptides

High molecular bands of type VI collagen were seen by silver staining in the *in vitro* control sample not exposed to proteases. These bands were abolished in the MMP-2 and MMP-9 cleaved samples, indicating that type VI collagen was degraded by these proteases (data not shown). Fragments of type VI collagen cleaved by MMP-2 or MMP-9 were identified with a statistically significant Mascot score (p<0.05). All protease-generated neo-epitopes were tested for homology. Among 184 MMP-2 or MMP-9 generated neo-epitopes five sequences were selected for immunizations since blasting showed that these sequences were unique to type VI collagen, and conserved throughout species:

1. 573'.YRGPEGPQGPPG'584; CO6A1 generated by MMP-2 or MMP-9
2. 1164'GIGIGNADIT.'1173; CO6A3 generated by MMP-2
3. 1164'.GIGIGNADIT'1173; CO6A3 generated by MMP-2
4. 2279'GPKGGIGNRG.'2288; CO6A3 generated by MMP-9
5. 2176'.LGPMGVPGRD'2185; CO6A3 generated by MMP-9

**Figure 1:**
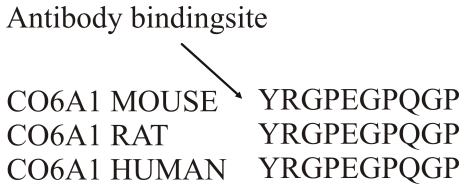
Alignment. Alignment of part of type VI collagen alpha 1 sequence for mouse, rat and human.

The sequence 573'.YRGPEGPQGPPG'584 (CO6-MMP) in the alpha 1 chain of type VI collagen generated by MMP-2 and MMP-9 was selected consequent to the best technical performance as the antibodies were able to distinguish between cleaved and uncleaved type VI collagen. In addition, this sequence is 100% homologous to human, rat and mouse (Figure 1).

### Clone characterization

The antibody with the best native reactivity, affinity and stability in the assay was chosen from the antibody-producing clones generated after the fusion between spleen cells and myeloma cells. The clone selected was determined to be the IgG1 subtype.

**Figure 2:**
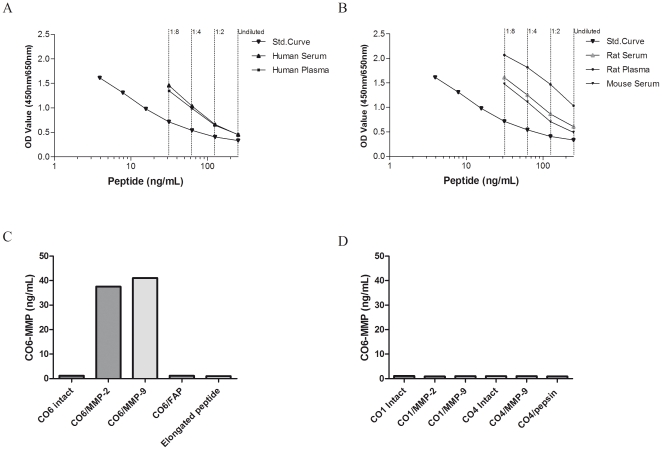
Assay characteristics. (A+B) ELISA run showing typical standard curves and native reactivity against (A) Human serum, plasma, (B) Rodent: Rat serum and plasma; mouse serum, plasma and urine. Native material was run undiluted, 1∶2, 1∶4, and 1∶8 as indicated (—).The signal is seen as the optical density at 450 nm, subtracting the background at 650 nm, as a function of peptide concentration; (C+D) Characterization of the CO6-MMP assay with regards to reactivity against (C) intact type VI collagen (CO6 intact), type VI collagen cleaved by MMP-2 (CO6/MMP-2), type VI collagen cleaved by MMP-9 (CO6/MMP-9), type VI collagen cleaved by fibroblast activation protein (FAP) (CO6/FAP), elongated peptide with extension of one amino acid at the neo-epitope site; (D) Intact type I collagen (CO1), type I collagen cleaved by MMP-2 (CO1/MMP-2), type I collagen cleaved by MMP-9 (CO1/MMP-9), intact type IV collagen (CO4), type IV collagen cleaved by MMP-2 (CO4/MMP-2), type IV collagen cleaved by MMP-9 (CO4/MMP-9), type IV collagen cleaved by pepsin (CO4/pepsin).

The clone was reactive to human serum and plasma (Figure 2A), rat serum and plasma (Figure 2B) and mouse serum (Figure 2B).

### Technical evaluation

The typical standard curve is presented in Figure 2A and 2B, showing a 4-parametric fit for the assay. The lower limit of detection (LDL) for the assay was 0.30 ng/mL. Dilution recovery was within 100±20% (Table 1). The inter- and intra-assay variation was a mean 4.0 and 10.1% respectively (Table 2). The analyte stability was acceptable for 2–10x freeze/thaw cycles within 100+/−20% (Table 3).

**Table 1:** Percentage dilution recovery for the CO6-MMP assay.

CO6-MMPng/mL	HS144.7	HS236.5	RS19.92	RS214.11
Undil	100%	100%	100%	100%
Dilution 1∶2	91%	87%	113%	109%
Dilution 1∶4	98%	90%	110%	105%
Dilution 1∶8	98%	86%	102%	96%
Dilution 1∶16	93%	92%	93%	101
**Mean**	**95%**	**89%**	**104%**	**103%**

HS = human serum; RS = rat serum.

**Table 2:** Inter- and intra-assay variation for the CO6-MMP assays using human serum quality control samples.

CO6-MMPSample	Amount(ng/mL)	Intra-assayvariability %	Inter-assayvariability %
HS1	7.6	4.5	10.1
HS2	11.7	4.8	13.3
HS3	10.5	2.9	8.7
HS4	13.1	4.3	9.4
HS5	13.8	3.7	9.2
HS6	13.3	4.2	10.5
HS7	17.8	3.8	9.8
HS8	21.3	4.3	9.9
**Mean**	**13.6**	**4.1**	**10.1**

The variation was calculated as the mean variation between 10 individual determinations of each sample.

**Table 3:** Analyte stability in three rat- and three human serum samples in 10 freeze/thaw cycles.

Analyte stability	Percent recovery compared to 1×freeze/thaw	
Freeze/thaw cycles	Human CO6-MMP	Rat CO6-MMP
2x	95.2	93.6
3x	86.1	96.0
4x	102.7	92.9
5x	105.7	103.6
6x	90.1	96.7
7x	98.1	99.2
8x	92.4	95.1
9x	91.4	99.7
10x	85.2	104.6

All data are shown as percent recovery compared to day 0 or 1 freeze/thaw cycle.

### ELISA characterization

To characterize the analytes detected in the assay, different collagens were cleaved with different proteases. Both MMP-2 and MMP-9 were able to generate the CO6-MMP fragment from collagen type VI (Figure 2C). In contrast, the fragment was not found in either intact or FAP-cleaved type VI collagen. Finally, no cross-reactivity was seen between CO6-MMP and intact or cleaved type I or IV collagens, which have high homology with the immunization sequence of type VI collagen (Figure 2D). No reactivity was seen against the elongated synthetic peptide, as expected proving neo-epitope reactivity (Figure 2C).

### Evaluations performed in the BDL study

During the 4 weeks, 3 of 40 rats, 2 BDL and 1 Sham, were put down due to excessive weight loss.

**Figure 3:**
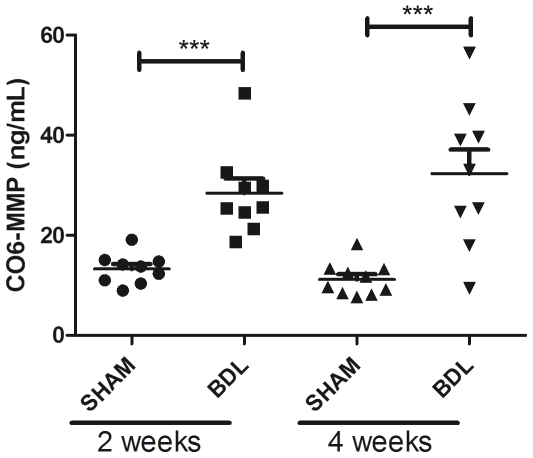
CO6-MMP serum levels in BDL-rats. BDL rat liver fibrosis model. Serum CO6-MMP was assessed in BDL and sham operated rats at termination. Termination time points were 2 and 4 weeks after surgery. Levels of CO6-MMP were significantly elevated in BDL rats compared to sham levels at both the two-( mean BDL 29.5 ng/mL, mean sham: 14.2 ng/mL, p = 0.0001) and four week mean BDL: 33.0 ng/ml, (mean sham: 11.8 ng/mL, p = 0.0003) termination point.

Levels of CO6-MMP were significantly elevated in BDL rats compared to sham levels at both the two-(mean BDL 29.5 ng/mL, mean sham: 14.2 ng/mL, p = 0.0001) and four week (mean BDL: 33.0 ng/ml, mean sham: 11.8 ng/mL, p = 0.0003) termination point (Figure 3).

**Figure 4:**
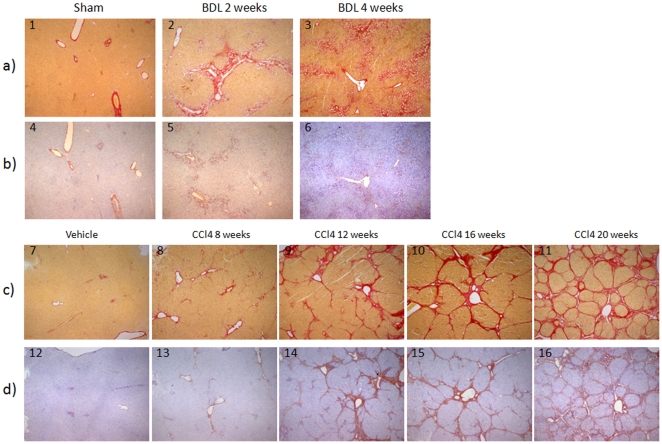
Type VI collagen in the liver. (A) Sirius Red photomicrographs showing the hepatic structure in rats 4 weeks after a sham operation (1), 2 weeks after BDL (2) and 4 weeks after BDL (3) (B) Immunohistochemical analysis of type VI collagen. Type VI collagen is localized around fibrotic structures. (C) Sirius Red photomicrographs showing the hepatic structure in rats 20 weeks after vehicle treatment (7), 8 weeks of CCl4 treatment (8), 12 weeks of CCl4 treatment (9), 16 weeks of CCl4 treatment (10) and 20 weeks of CCl4 treatment (11). (D) Immunohistochemical analysis of type VI collagen. Type VI collagen is localized around the fibrotic bands. Original magnification ×40.

By immunohistochemistry, collagen type VI deposition was found exclusively in the venous wall of healthy rats (Figure 4B). In contrast, in BDL rats in which marked ductal proliferation was seen around the portal tract with the formation of multiple neo-bile ducts, more extensive type VI collagen was found (Figure 4B).

### Evaluations performed in the CCL4 study

**Figure 5:**
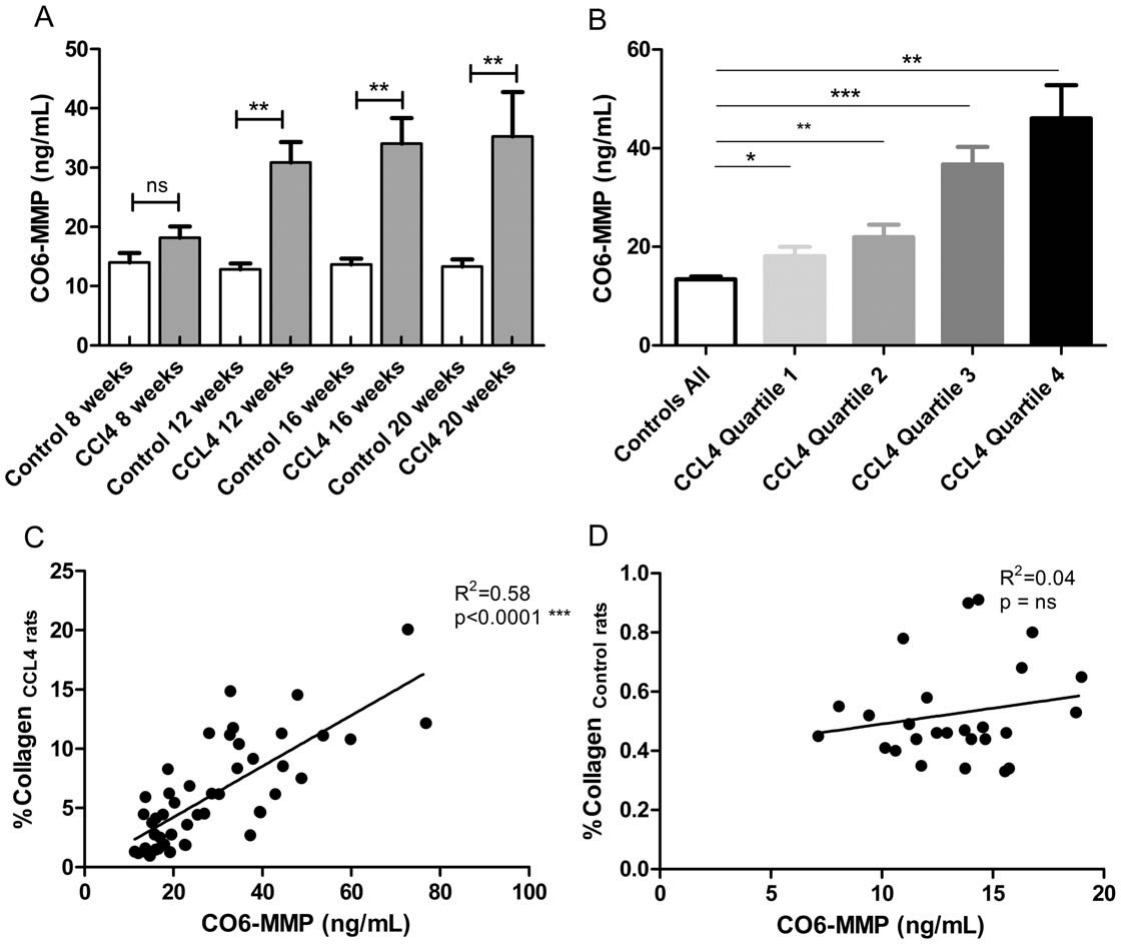
CO6-MMP serum levels in CCl4-treated rats. CCl4 rat liver fibrosis model: (A) Serum CO6-MMP was assessed in control rats at termination (controls) as well as in CCl4-treated rats at termination (CCl4) at weeks 8, 12, 16, 20. Results shown are mean ± standard error of the mean (SEM); (B) Serum CO6-MMP in all controls pooled and CCl4 rat stratified in quartiles according to total collagen in the liver; (C) Correlations between CO6-MMP and Sirius red in (C) CCl4 rats and in (D) control rats; Asterisks indicate statistical significance as indicated by bars. (** = p<0.05; *** = p<0.001, ns = non-significant difference).

In the CCL4 rat model, levels of CO6-MMP were significantly elevated at all time points compared to baseline levels, except at week 8 (week 12: mean CCl4 30.9 ng/mL, mean control 12.8 ng/mL, p = 0.0015; week 16: mean CCl4 34.0 ng/mL, mean control 13.7 ng/mL, p = 0.0018, week 20: mean CCl4 35.3 ng/mL, mean control 13.3 ng/mL, p = 0.0033) (Figure 5A). When CCl4- treated rats were classified by the total amount of collagen in the liver evaluated by histology (Sirius Red) (Figure 5B), it clearly seen that the marker was elevated in the lowest quartile of total collagen (quartile 1), as well as in quartiles 2–4 compared to control animals (mean Q1: 18.1 ng/mL, p>0.03; mean Q2: 22.0 ng/mL, p<0.05; mean Q3: 36.7 ng/mL, p<0.0001; mean Q4: 46.1 ng/mL, p = 0.0018; mean across all controls 13.4 ng/mL). The correlation between CO6-MMP and total collagen was highly significant in CCl4-treated rats (p<0.0001, R^2^ = 0.58) (Figure 5C), however this was not seen in control rats (p = ns, R^2^ = 0.04) (Figure 5D).

By immunohistochemistry, collagen type VI deposition was found exclusively in the venous wall of control rats (Figure 4D). In contrast, in CCl4-treated rats type VI collagen was located along the fibrotic bands (figure 4D).

## Discussion

This is, to our knowledge, the first study to present the development of an assay specific for a fragment of type VI collagen generated by MMPs. Our main findings were: 1) A technically robust assay was developed with monoclonal antibodies highly specific for the CO6-MMP fragment. The assay had acceptable inter-, and intra-assay variation, dilution recovery and a low limit of detection; 2) CO6-MMP levels were assessed in two different animal models of liver fibrosis: CCl4 and BDL. In both models we found significant increased levels in liver fibrotic rats compared to controls. In addition CO6-MMP levels were significantly correlated to collagen deposition in the CCl4 model of liver fibrosis.

ECM remodeling is an essential part of tissue homeostasis. Extensive ECM remodeling is associated with a range of pathologies [7], [8], [20], [26], [32], [33] in which fibrosis is of particular relevance. Biochemical markers consisting of protein fragments from pathologic tissue remodeling may be useful for diagnostic and prognostic purposes [34]. Such an approach focusing on neo-epitopes have primarily been useful for the arthritis and bone field [35], and for liver fibrosis in monitoring degradation of collagen type III [36], [37].

The gelatinases MMP-2 and MMP-9 have been investigated and documented to be highly regulated in fibrous tissue [12], [13]. Cleavage of type VI collagen with these two proteases generated several fragments. Among these CO6-MMP was chosen due to it being unique and conversed among species. Several assays already exist for measuring total type VI collagen in different pathologies, using polyclonal antibodies (antibodies-online GmbH) or monoclonal antibodies (Uscn Life Science). Circulating levels of total type VI collagen patients with alcoholic liver disease have been investigated by Stickel *et al* [24], who found an significant elevation in alcoholic patients compared to controls. In addition they concluded that total collagen type VI already waselevated in early fibrotic states and therefore seem to be an important indicator of early fibrotic transformation [24]. These data are consistent with our data indicating CO6-MMP is an early marker for fibrosis and they are in alignment with the fact that collagen type VI may be a good candidate biomarker. The CO6-MMP assay provides additional information on protease mediated tissue destruction in face of the total collagen markers. Such a careful deconstruction of the information entailed in the precise analyte may prove important to understand the processes that are leading to increased tissue formation as well as tissue degradation, and therefore eventually resolution of diseases. This may be assisted by the measurement of protein degradation and protein formation, in face of a crude measure of total protein [11], [36], [38]. The competitive ELISA was technically stable with a acceptable dilution recovery, as well as inter- and intra-variation for all matrices tested. The highest sensitivity for the assay was observed in the range of 4–125 ng/mL. Characterization of the selected monoclonal antibody revealed strong reactivity towards human, mouse and rat serum as well as the CO6-MMP peptide, strongly suggesting that the antibody recognizes this amino acid sequence for type VI collagen in native samples in complicated matrices. Characterizations using the final ELISA format showed that the recognized peptide fragment was generated by MMP-2 and MMP-9. Furthermore, it was seen that the antibody was specific against the neo-epitope generated in type VI collagen, as no response was detected when type I or type IV collagen was cleaved by MMP-2 and -9. In addition the antibody did not recognise the elongated peptide, indicating neo-epitope specificity. The analyte stability was very good for both human- and rat serum CO6-MMP all recoveries within 100+/−20%.

It is well-appreciated that the BDL and the CCl4 models describe two different fibrotic processes in which increased ECM remodelling and excessive collagen deposition are key characteristics. The CO6-MMP was significantly related to liver fibrosis in CCL4 treated rats treated for 12–20 weeks. Furthermore, when rats were classified into quartiles according to the extent of fibrosis defined as the amount of collagen in the liver, we observed that the marker was elevated in all quartiles. The marker also correlated highly significantly to total collagen in the livers of CCL4-treated rats; however this was not the case in control rats, strongly indicating liver specific pathological relevance of the neo-epitope.

In the BDL model of liver fibrosis, serum CO6-MMP was elevated 2 and 4 weeks after BDL surgery compared to baseline and sham levels. These data are in agreement with the previous studies highlighting that type VI is generated during fibrogenesis by the activated hepatic stellate cells in the liver [2], [37] and that MMP levels become elevated and unbalanced during fibrosis [1]. These data suggest that liver fibrosis is a high turnover disease, not may nor exclusively be described as an accumulation disease with increased collagen formation. Additionally, our data are in alignment investigating the collagen turnover profile in fibrotic rats, which demonstrated that a MMP-9 generated fragment of collagen type III (CO3-610) was elevated in the BDL rat model [37]. These combined data, past and present, indicate that liver fibroses may be a high collagen turnover disease with both increased collagen formation [23], [38] and collagen degradation. This further emphasis the need of measuring the individual processes, enabling discovery of pathways leading to a resolution of the disease – by lowering tissue formation and increasing tissue degradation transiently. Such an investigation may not be obtained by exclusively measuring the total protein levels.

The systemic level of a biochemical marker is the result of the activity level and number/area of affected tissues. As such, levels of the CO6-MMP analyte are a systemic measurement of several local events. Type VI collagen has been identified within most tissues in different quantities [16]. In addition chronic liver diseases are associated with co-morbidities like osteoporosis, protein and calorie malnutrition [39], which may cause increased CO6-MMP levels originating from secondary tissues. The increased CO6-MMP levels in the presented two animal models may primarily derive from fibrosis in the liver but there may be confounded by effects from other tissues e.g. bone loss or muscular dystrophy, albeit bone only contain minute quantities of type IV collagen [39]. The exact contribution to the systemic pool of CO6-MMP from different tissues, healthy and disease affected remains to be more carefully investigated.

This study carries some limitations. One major limitation of this study is that it is carried out in homogeneous, inbred laboratory rats with a synchronous induction of liver disease, which that bear little resemblance to the highly complicated presentation of clinical description of liver fibrosis. Further investigations in clinical settings are needed to provide more information on the usefulness of CO6-MMP.

In conclusion, we have developed an assay using a specific monoclonal antibody for the detection of CO6-MMP, a collagen type VI fragment generated by MMP-2 and -9. It was demonstrated that this marker was elevated in two pre-clinical models of fibrosis, the BDL and the CCl4 rat model, indicating that there is a high potential for the use of neo-epitope biomarkers in ECM-related diseases.
